# Supplementary material for: Estimation of the Periodontal Inflamed Surface Area by Simple Oral Examination
Source: J Clin Med. 2021 Feb 12;10(4):723. doi: 10.3390/jcm10040723 (PMC7917734; doi:10.3390/jcm10040723)
Supplement: Supplementary file 1 [file jcm-10-00723-s001.pdf]

Supplemental materials

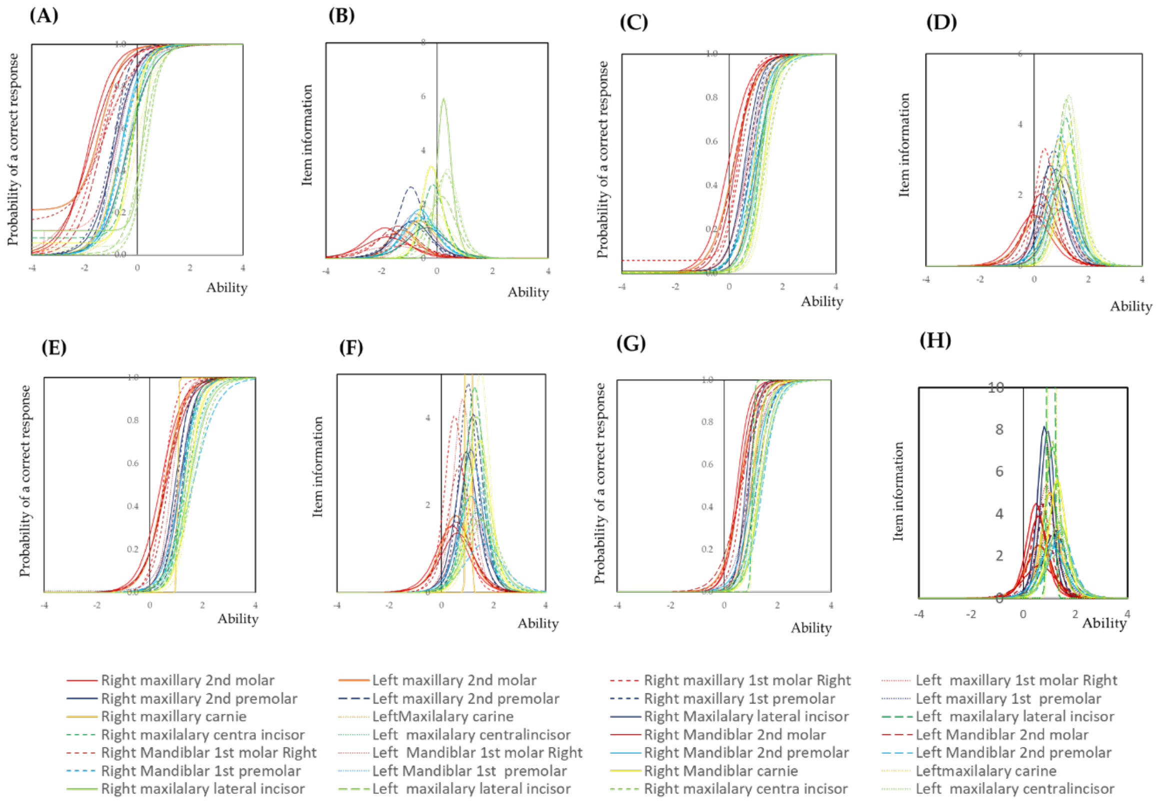

Figure S1. Item response curves and item information curves of the number of bleeding sites.

(A), (B): 1 site in each tooth; (C), (D) 2 sites in each tooth; (E), (F): 3 sites in each tooth; (G), (H): 4 sites in each tooth. In each group, the curves on the left are item-response curves and those on the right are item-information curves.

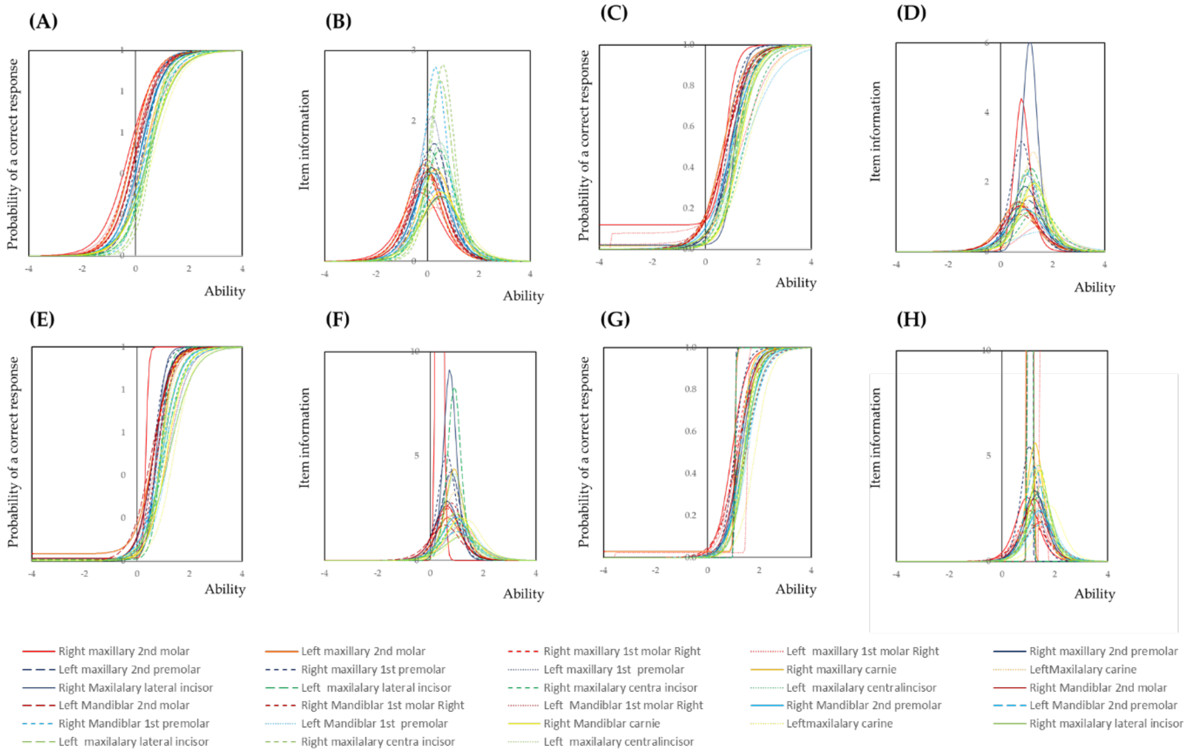

Figure S2. Item-response curves and item-information curves for the maximum value of probing depth.

(A), (B): 1 site in each tooth; (C), (D): 2 sites in each tooth; (E), (F): 3 sites in each tooth; (G), (H): 4 sites in each tooth. In each group, the curves on the left are item-response curves and those on the right are item-information curves.

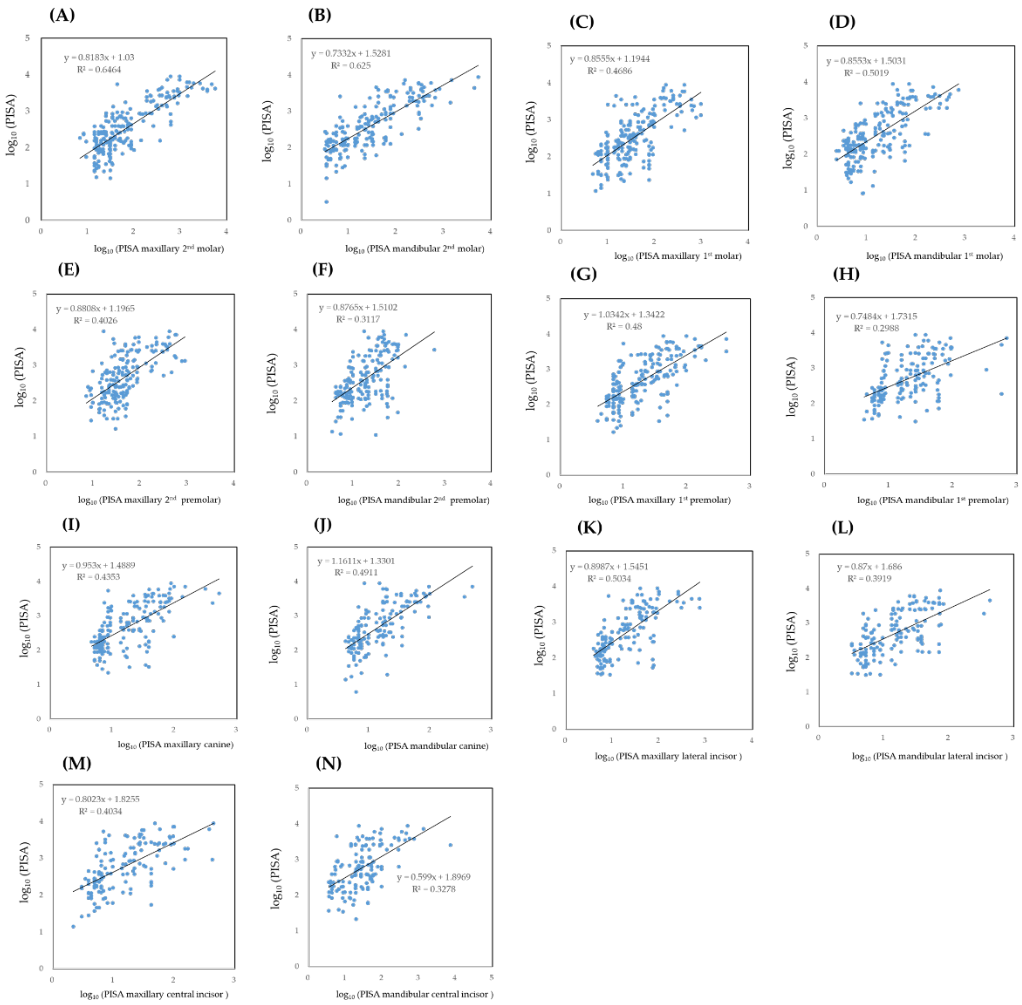

Figure S3. Scatter plot of PISA against the PISA of each tooth type.

For the scatterplot, PISA was $\log_{10}$ transformed. (A) Maxillary second molar, (B) mandibular second molar, (C) maxillary first molar, (D) mandibular first molar, (E) maxillary second premolar, (F) mandibular second premolar, (G) maxillary first premolar, (H) mandibular first premolar, (I) maxillary canine, (J) mandibular canine, (K) maxillary lateral incisor, (L) mandibular lateral incisor, (M) maxillary central incisor, (N) mandibular central incisor. The values of zero were eliminated from the plot.

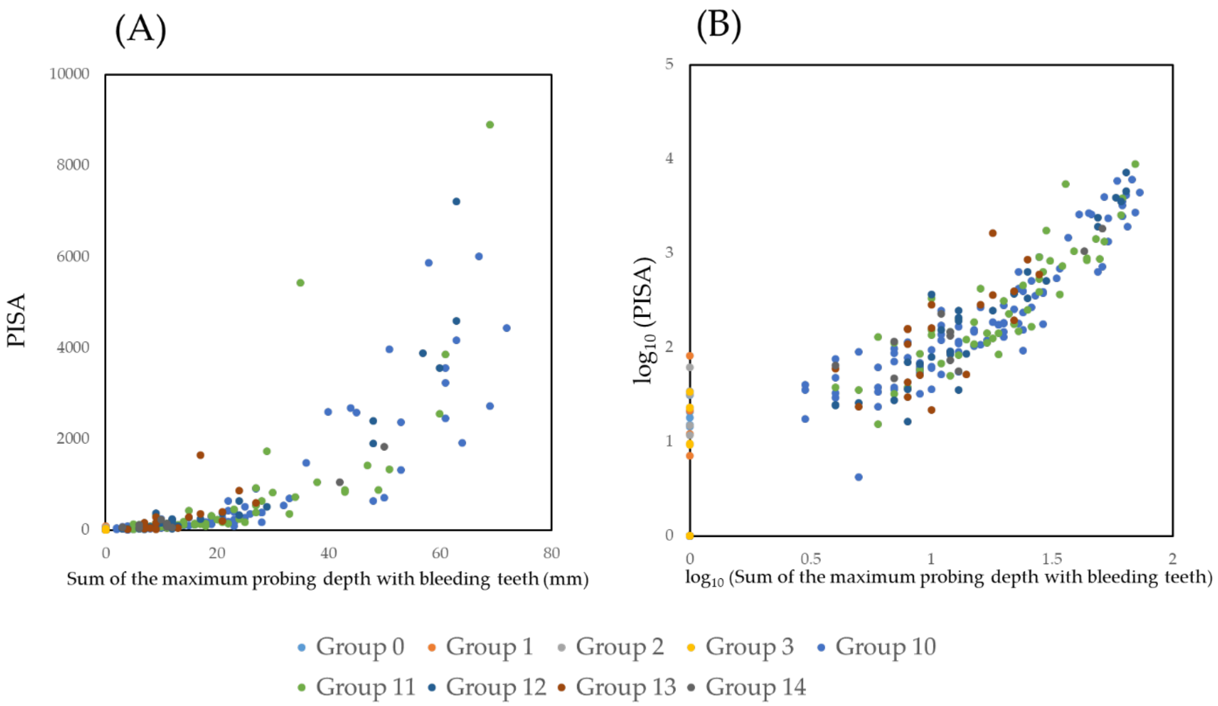

Figure S4. Scatter plot of PISA against the PISA of each tooth type.

The data shown in Figure 4 were categorized by the groups to elucidate the effect of missing teeth on PISA. (A) Scatter plot by net values; (B) scatter plot by log$_{10}$-transformed values. Group 0: no missing teeth and no bleeding in 10 teeth, Group 1: one missing tooth and no bleeding in nine teeth, Group 2: two missing teeth and no bleeding in eight teeth, Group 3: three missing teeth and no bleeding in seven teeth. Group 10: one missing tooth in 10 teeth, Group 11: one missing tooth in 10 teeth, Group 12: two missing teeth in 10 teeth, Group 13: three missing teeth in 10 teeth, Group 14: Four or five missing teeth in 10 teeth.

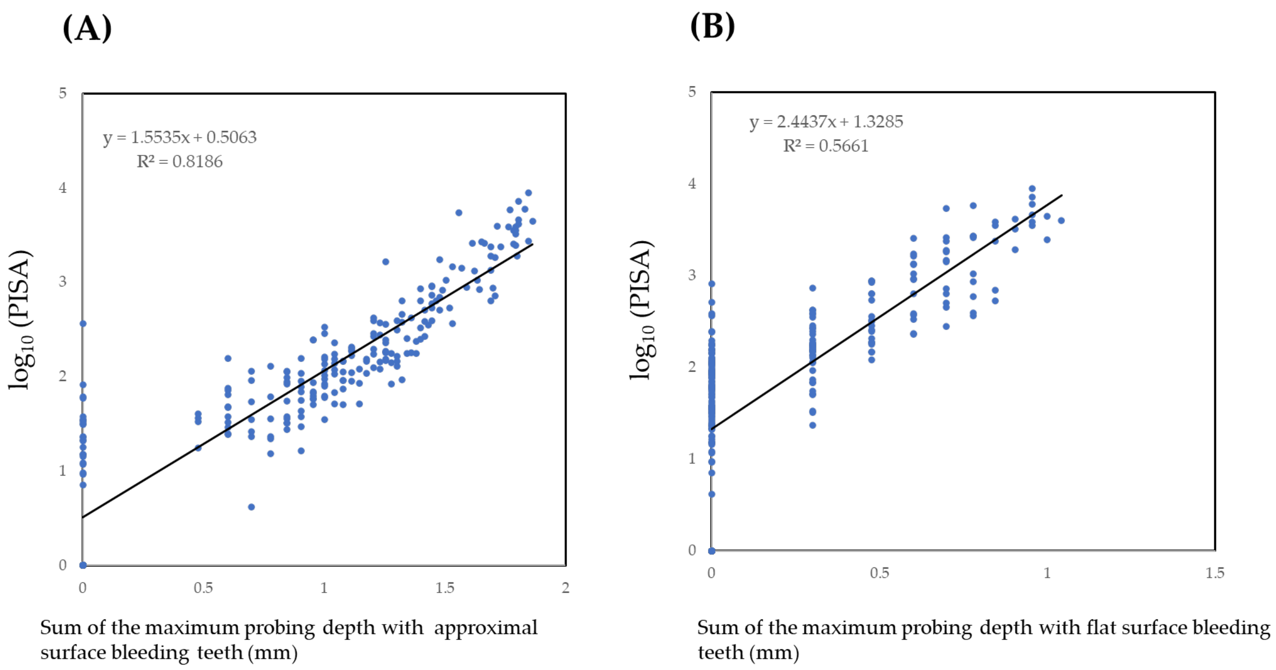

Figure S5. Scatter plot of PISA against the PISA calculated by bleeding with approximal surface (A) and flat surface (B)

Similarity, prediction of PISA was carried out by the at least bleeding of approximal surface or flat surface in one site with in one teeth. When compared R^2 of the regressions, R^2 of regression by approximal surface was higher than that of flat surface. It indicates that the effect for PISA value by approximal surface was higher than that of flat surface.

Table S1. Three-parameter logistic model for number of bleeding sites

			1 site			2 sites			3 sites			4 sites		
			Discrimination	Difficulty	Guessing	Discrimination	Difficulty	Guessing	Discrimination	Difficulty	Guessing	Discrimination	Difficulty	Guessing
Maxillary	2nd molar	Right	1.75	-0.12	0	28.99	0.64	0.136	3.22	1.10	0	42.04	0.67	0.03
		Left	2.28	0.02	0	2.54	0.40	0	2.50	1.50	0.043	32.86	0.68	0.03
	1st molar	Right	2.05	-0.01	0	2.65	0.47	0	2.53	1.44	0	31.88	0.68	0.03
		Left	1.56	-0.03	0	2.08	0.65	0.074	2.04	1.51	0	27.94	0.68	0.02
	2nd premolar	Right	2.40	0.33	0	3.04	0.65	0	2.52	1.47	0	22.12	0.69	0.01
		Left	2.34	0.45	0	2.61	0.74	0	4.76	1.59	0	18.81	0.69	0.00
	1st premolar	Right	2.52	0.25	0	3.79	0.54	0	4.29	1.44	0	33.36	0.67	0.01
		Left	2.61	0.35	0	3.26	0.69	0.015	3.34	1.67	0	18.57	0.71	0.00
	Canine	Right	2.21	0.52	0	2.63	0.83	0.014	3.39	1.78	0.008	18.05	0.71	0.00
		Left	2.30	0.50	0	3.43	0.94	0.012	3.07	1.79	0	18.31	0.70	0.01
	Lateral incisor	Right	1.84	0.69	0	4.70	0.82	0.017	6.02	1.61	0.016	17.96	0.72	0.01
		Left	2.71	0.63	0	3.42	0.87	0	4.57	1.77	0.005	94.13	0.69	0.00
	Central incisor	Right	2.09	0.77	0	2.38	1.16	0	2.18	1.96	0	23.30	0.72	0.00
		Left	2.47	0.68	0	2.96	0.95	0	3.08	1.83	0	15.79	0.73	0.01
Mandibular	2nd molar	Right	2.01	0.17	0	2.48	0.49	0	2.61	1.43	0	18.33	0.69	0.01
		Left	1.94	0.20	0	2.30	0.48	0	2.24	1.32	0	28.23	0.68	0.04
	1st molar	Right	2.24	0.13	0	2.53	0.49	0	2.34	1.67	0	14.67	0.73	0.01
		Left	1.88	0.04	0	2.45	0.63	0.023	2.40	1.59	0	24.31	0.68	0.02
	2nd premolar	Right	2.12	0.35	0	2.47	0.67	0	2.54	1.81	0	16.06	0.73	0.01
		Left	1.84	0.58	0	2.32	0.83	0	2.00	1.88	0	18.15	0.70	0.00
	1st premolar	Right	3.23	0.50	0	3.59	0.71	0	2.18	1.77	0	19.24	0.73	0.02
		Left	1.78	0.68	0	1.63	1.14	0	2.49	2.12	0.003	14.41	0.76	0.01
	Canine	Right	1.91	0.70	0	3.02	0.98	0	2.73	1.99	0	23.76	0.72	0.00
		Left	3.20	0.94	0.066	2.18	1.25	0	2.26	2.40	0.004	175.09	0.69	0.01
	Lateral incisor	Right	1.75	0.71	0	2.30	0.84	0	1.86	2.12	0	22.31	0.72	0.00
		Left	2.99	0.70	0	3.34	0.74	0	2.79	1.82	0.001	29.81	0.71	0.00
	Central incisor	Right	3.01	0.81	0	2.94	0.93	0	2.40	1.85	0	17.17	0.72	0.01
		Left	2.31	0.74	0	2.61	0.93	0	2.02	1.97	0	11.42	0.76	0.01

Table S2. Three-parameter logistic model for the maximum value of probing depth

			Maximum probing depth											
			3 mm			4 mm			5 mm			6 mm		
			Discrimination	Difficulty	Guessing	Discrimination	Difficulty	Guessing	Discrimination	Difficulty	Guessing	Discrimination	Difficulty	Guessing
Maxillary	2nd molar	Right	2.10	-2.08	0.00	2.57	-0.29	0.00	3.21	0.62	0.05	51.96	0.66	0.08
		Left	2.53	-1.61	0.19	2.51	-0.09	0.00	2.99	0.66	0.00	41.41	0.66	0.07
	1st molar	Right	2.13	-1.67	0.00	3.95	0.04	0.05	4.01	0.70	0.02	43.18	0.66	0.07
		Left	2.29	-1.58	0.00	2.87	-0.03	0.00	3.44	0.72	0.00	42.20	0.66	0.07
	2nd premolar	Right	2.34	-1.08	0.00	3.34	0.27	0.00	3.48	1.11	0.00	22.41	0.65	0.01
		Left	3.30	-1.13	0.00	3.48	0.48	0.00	4.31	1.35	0.00	35.76	0.71	0.01
	1st premolar	Right	2.40	-1.16	0.00	3.88	0.39	0.01	4.61	1.17	0.00	19.43	0.66	0.01
		Left	2.70	-0.98	0.00	3.10	0.33	0.00	2.85	1.24	0.02	23.95	0.69	0.03
	Canine	Right	2.44	-0.83	0.00	4.14	0.59	0.00	5.51	1.27	0.00	4.67	0.75	0.00
		Left	2.90	-0.63	0.00	3.34	0.72	0.00	2.75	1.46	0.01	41.12	0.69	0.01
	Lateral incisor	Right	2.19	-0.59	0.00	3.39	0.72	0.00	3.32	1.30	0.00	5.53	0.69	0.00
		Left	3.14	-0.51	0.00	4.39	0.82	0.01	4.14	1.44	0.00	35.13	0.70	0.00
	Central incisor	Right	2.31	-0.54	0.00	2.63	0.67	0.00	3.08	1.53	0.01	5.67	0.82	0.00
		Left	2.38	-0.61	0.00	3.76	0.72	0.00	3.34	1.48	0.00	4.65	0.86	0.00
Mandibular	2nd molar	Right	1.87	-1.95	0.00	3.14	-0.03	0.00	2.61	0.73	0.00	48.46	0.65	0.06
		Left	2.26	-1.58	0.00	2.87	-0.10	0.00	2.46	0.71	0.00	3.13	0.42	0.00
	1st molar	Right	1.63	-1.44	0.15	3.51	0.10	0.00	3.69	0.82	0.00	31.11	0.64	0.03

	Left	1.40	-1.70	0.00	3.08	0.37	0.00	4.62	0.94	0.00	16.98	0.66	0.01
2nd premo-lar	Righ t	2.60	-0.83	0.00	3.22	0.52	0.00	3.10	1.27	0.00	17.11	0.70	0.01
	Left	2.37	-0.82	0.00	2.78	0.67	0.00	1.87	1.85	0.00	4.59	1.07	0.00
1st premo-lar	Righ t	2.56	-0.77	0.00	4.19	0.63	0.00	3.20	1.42	0.00	4.33	0.88	0.00
	Left	2.46	-0.61	0.00	3.25	0.82	0.00	2.62	1.66	0.00	5.19	0.94	0.00
Canine	Righ t	3.88	-0.44	0.06	3.72	0.95	0.00	3.29	1.65	0.00	5.53	0.95	0.00
	Left	2.71	-0.23	0.04	3.98	1.10	0.00	4.31	1.66	0.00	28.09	0.75	0.00
Lateral in-cisor	Righ t	4.32	-0.07	0.09	3.52	0.77	0.00	2.25	1.71	0.00	4.50	1.02	0.00
	Left	3.01	-0.06	0.00	4.24	0.86	0.00	3.63	1.55	0.00	5.71	0.82	0.00
Central in-cisor	Righ t	3.54	0.09	0.00	3.03	1.03	0.00	2.46	1.81	0.00	15.60	0.78	0.02
	Left	3.44	0.12	0.03	4.34	0.96	0.00	3.12	1.72	0.00	3.81	1.08	0.00

Table S3: Quick reference predictive PISA values

Sum of maximum value of probing depth with bleeding of selected teeth	Predicted PISA	Sum of maximum value of probing depth with bleeding of selected teeth	Predicted PISA	Sum of maximum value of probing depth with bleeding of selected teeth	Predicted PISA
0	3	20	304	40	915
1	3	21	328	41	952
2	8	22	353	42	989
3	15	23	379	43	1027
4	23	24	406	44	1065
5	33	25	433	45	1104
6	45	26	461	46	1143
7	57	27	489	47	1183
8	71	28	519	48	1223
9	85	29	548	49	1264
10	101	30	579	50	1305
11	117	31	610	51	1347
12	135	32	642	52	1390
13	153	33	674	53	1432
14	172	34	707	54	1476
15	192	35	740	55	1519
16	213	36	774	56	1564
17	234	37	808	57	1608
18	257	38	843	58	1653
19	280	39	879	59	1699
